# Diffuse Facial Leukoderma Secondary to Localized Use of Hydroquinone

**DOI:** 10.7759/cureus.67751

**Published:** 2024-08-25

**Authors:** Christina Artz, Mavra Masood, Tasneem F Mohammad

**Affiliations:** 1 Dermatology, Henry Ford Health, Detroit, USA

**Keywords:** melasma, acquired hypopigmentation, depigmentation, leukoderma, hydroquinone

## Abstract

Disorders of hyperpigmentation are extremely common, and hydroquinone remains one of the most common treatments for hyperpigmentation. Adverse events reported with hydroquinone use include acneiform eruptions, ochronosis, and irritant dermatitis; leukoderma has been reported in rare instances. Largely, these cases report leukoderma localized to the site of application. However, we report a case of diffuse facial leukoderma with only localized use of hydroquinone. With appropriate and prompt treatment, this leukoderma can respond to vitiligo treatment algorithms.

## Introduction

Disorders of hyperpigmentation are prevalent, especially in skin of color. Worldwide, hydroquinone remains one of the most common treatments for disorders of hyperpigmentation, specifically melasma, post-inflammatory hyperpigmentation, and dyschromia from photoaging [1]. Adverse reactions with topical hydroquinone application include irritant or allergic dermatitis with short-term application and exogenous ochronosis, which occurs from chronic exposure [1]. However, in rare instances, it has been reported to cause leukoderma [2]. Here, we discuss a case of diffuse facial leukoderma after localized use of hydroquinone. This case report was presented as an oral presentation at the Cosmetic Surgery Forum on December 2, 2022, in Nashville, TN, USA. 

## Case presentation

**Figure 1:**
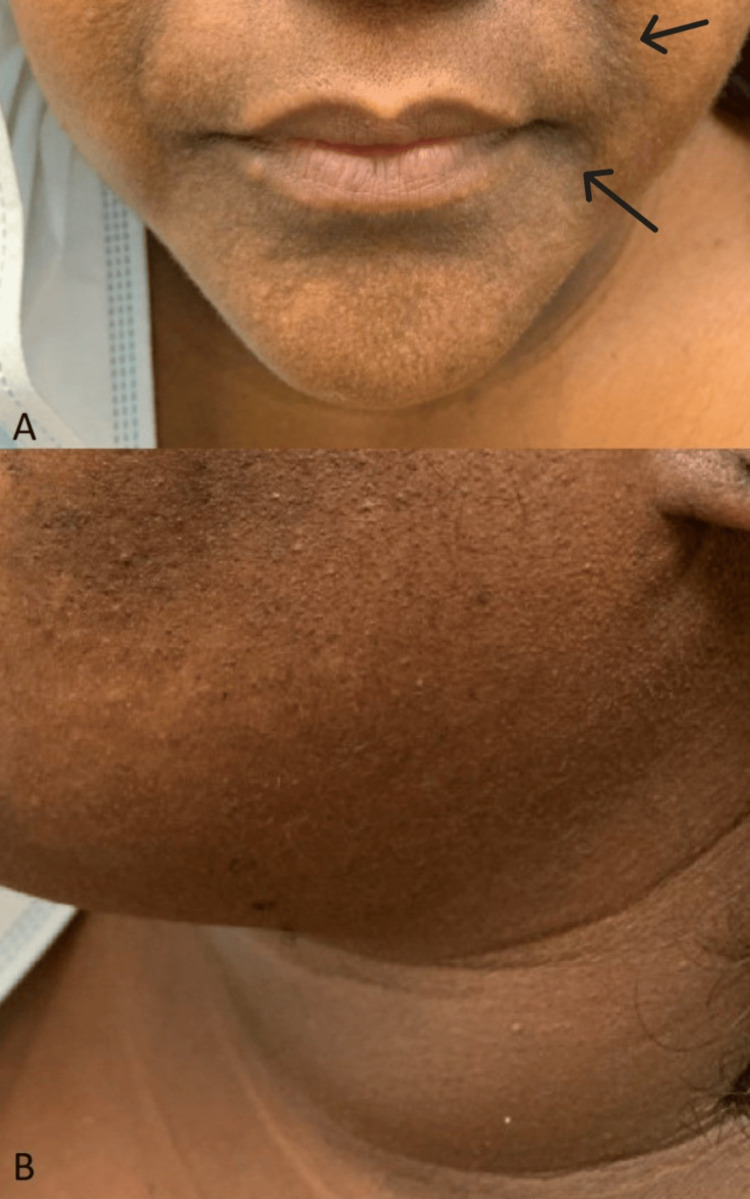
Facial hyperpigmentation A: Symmetric hyperpigmented patches on cheeks, nasolabial folds, and around the mouth present on the patient's initial visit; B: Demonstrating no involvement of the neck

A 48-year-old African American female with a history of prediabetes, hypertension, and hyperlipidemia presented with asymptomatic darkening around the eyes, mouth, and cheeks that had been ongoing for several years. Previous treatments included hydroquinone 2%, used intermittently without any results. The clinical exam demonstrated symmetric hyperpigmented patches only around the eyes, cheeks, and mouth (Figure 1). The patient was diagnosed with melasma. Treatment options were discussed, and the patient preferred to start a compounded hydroquinone 8%, tretinoin 0.1%, and fluocinolone 0.1% cream nightly versus hydroquinone 4% alone. Strict photoprotection with a broad-spectrum tinted mineral sunscreen was advised. The patient was counseled to stop using the compounded hydroquinone after three months and to follow up.

Seven months later, she presented to the clinic with diffuse lightening of her forehead, temples, nose, central face, and neck, specifically in areas where she had not been applying the compounded hydroquinone cream. This developed after one month of use. The patient stopped using the compounded cream but continued to note depigmentation. She denied any personal or family history of thyroid disease, vitiligo, or other autoimmune disease.

**Figure 2:**
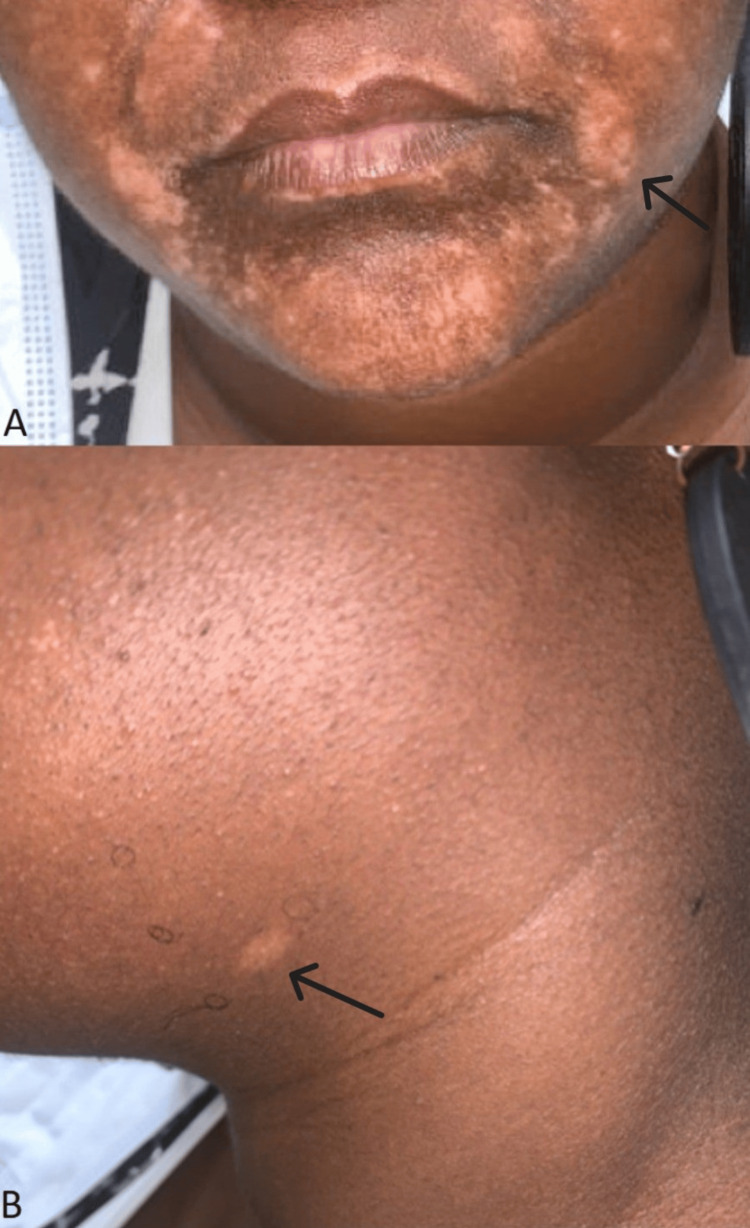
Hypopigmentation of the face and neck A: Depigmented macules and patches without scale or erythema that formed after treatment with the hydroquinone compounded topical; B: The areas shown along with the hypopigmented patches on the neck were not treated with the hydroquinone compound.

On exam, there were several hypopigmented to depigmented macules on her cheeks, chin, upper cutaneous lip, and lateral neck (Figure 2). A 4 mm punch biopsy was taken from a depigmented lesion from the left neck, which showed superficial perivascular dermatitis with dermal melanophages most consistent with post-inflammatory pigmentary alteration. The patient was started on pulse dexamethasone 4 mg on weekends for eight weeks, topical hydrocortisone 2.5% ointment twice daily, and natural sunlight exposure for 15 to 20 minutes daily. The patient noted a gradual resolution of depigmentation over the course of several months.

## Discussion

Hydroquinone has been used as a skin-lightening product for over 50 years, and 10 to 15 million tubes of hydroquinone-based products are sold annually [3]. Common side effects of hydroquinone include erythema and irritant dermatitis, while exogenous ochronosis typically occurs with prolonged use of higher concentrations [3]. Rare instances of chemical-induced leukoderma have been reported [2,4,5].

The first published case of persistent leukoderma was reported in 1982 with the use of 2% hydroquinone [6]. Since then, additional cases have been reported [2,4-6]. Exposures occurred in both cosmetic and occupational settings and the duration of use ranged from a few days to 15 months. Concentrations of hydroquinone varied between 0.06% and 7%, with the most common concentrations being 2% to 4%. Leukoderma was most commonly reported in African, American, and Indian populations [2,4-6].

In most reported cases, depigmentation was localized to areas that came into direct contact with hydroquinone. However, similar to our patient, another case reported progressive hypopigmented lesions after the application of 3% hydroquinone to the face that spread to sites distant from the application, including the scalp and trunk. While the mechanism of hydroquinone-induced leukoderma in this case remains unknown, the spread of leukoderma to untreated areas may suggest hydroquinone as a possible trigger of vitiligo in select patients [4].

The compounded cream also contained retinoic acid 0.1% and fluocinolone 0.1%, which we believe are less likely to have caused leukoderma. Retinoic acid has been shown to improve the efficacy of hydroquinone by increasing epidermal penetration [7]. Tretinoin may be used as monotherapy for melasma; however, it requires 20 to 40-week courses, making it less likely to have caused leukoderma in our patient, who had only been using it for one month. The corticosteroid component of the compounded cream reduces the irritation caused by retinoic acid [7]. While atrophy and hypopigmentation are reported side effects of long-term use of corticosteroids, topical corticosteroids may be used in the management of post-inflammatory hypopigmentation, though the mechanism is unclear [7]. 

## Conclusions

Leukoderma is often the intended effect of the monobenzyl ether form of hydroquinone, but it is not the intended effect of hydroquinone. It is important to remember this rare, but reported, side effect of hydroquinone when counseling patients, given that even low concentrations can induce leukoderma. If leukoderma does occur, treatment following standard vitiligo algorithms can be used to achieve improvement.

## References

[1] Shivaram K, Edwards K, Mohammad TF (2024). An update on the safety of hydroquinone. Arch Dermatol Res.

[2] Jow T, Hantash BM (2014). Hydroquinone-induced depigmentation: case report and review of the literature. Dermatitis.

[3] Tse TW (2010). Hydroquinone for skin lightening: safety profile, duration of use and when should we stop?. J Dermatolog Treat.

[4] Das A, Ghosh A, Kumar P (2019). Chemical leukoderma due to hydroquinone: an unusual phenomenon. Indian J Dermatol Venereol Leprol.

[5] Atzori L, Zanniello R, Sarais G (2018). Disfiguring leukoderma caused by banned cosmetics: a quiz. Acta Derm Venereol.

[6] Fisher AA (1982). Leukoderma from bleaching creams containing 2% hydroquinone. Contact Dermat.

[7] Halder RM, Richards GM (2004). Management of dyschromias in ethnic skin. Dermatol Ther.

